# Positive Childhood Experiences and Spiritual Well-Being: Psychological Flexibility and Meaning-Based Coping as Mediators in Turkish Sample

**DOI:** 10.1007/s10943-024-02079-4

**Published:** 2024-06-24

**Authors:** Sibel Maral, Huzeyfe Bilmez, Seydi Ahmet Satici

**Affiliations:** Department of Psychological Counselling, Faculty of Education, Yildiz Technical University, Istanbul, Türkiye

**Keywords:** Spiritual well-being, Positive childhood experiences, Psychological flexibility, Meaning-based coping

## Abstract

Spiritual well-being enhances life quality, acts as a stress reliever, and mitigates unfavorable feelings. It helps individuals find meaning and purpose, increasing inner peace and happiness while improving stress management and overall well-being. This study examined whether positive childhood experiences are linked to spiritual well-being and if psychological flexibility and meaning-based coping serve as mediators. The sample included 1061 participants (Mage = 39.38; SD = 8.82) from various Turkish cities. Structural equation modeling assessed relationships between spiritual well-being, positive childhood experiences, psychological flexibility, and meaning-based coping. Results showed that positive childhood experiences directly enhance spiritual well-being, mediated by psychological flexibility and meaning-based coping. This underscores the significance of fostering positive childhood experiences to promote spiritual well-being and coping mechanisms.

## Introduction

The World Health Organization (WHO) expanded its health definition to include spirituality, recognizing its significance alongside social, mental, and physical well-being (WHO, 1998). Spirituality, a multifaceted aspect of human existence, involves seeking meaning, forming connections beyond oneself, and enhancing well-being through various practices (Carmo, 2022; Korac-Kakabadse et al., 2002; Lalani, 2020). It encompasses purpose, self-awareness, and interconnectedness, promoting inner peace and spiritual well-being (Coyle, 2002). Strong spiritual bonds enhance life satisfaction and aid in coping (Delgado, 2005), reducing negative emotions and psychological distress while fostering resilience, quality of life and spiritual well-being (Brown et al., 2013; Fehring et al., 1987).

Spiritual well-being pertains to the quality of an individual's interactions with others, themselves, nature, and/or a divine entity (Fisher, 2008). It embodies acceptance, integration, and a profound sense of completeness, encapsulating the core essence of human existence (Hamilton et al., 2017). Spiritual well-being, defined by positive attributes such as optimism, inner tranquility, and unconditional love, is associated with improved psychosocial outcomes, decreased substance use, and promotes emotional stability, generativity, and overall well-being, all of which are beneficial aspects of both children and adult development (Bharti & Verma, 2018; Fisher, 2015; Hammermeister & Peterson, 2001; Zubairi & Sawari, 2018) and it intersects with concepts such as well-being, meaning, connections, belonging, safety, and environmental consciousness (Kreitzer, 2012).

According to a systematic study by Unterrainer et al. (2012), spiritual well-being is essential to the healing process from mental health conditions and offers protection against addiction or self-harming behaviors. Additionally, Song et al. (2016) suggest that early life experiences, such as childhood emotional neglect, lead to low spiritual well-being, while the presence of spiritual well-being serves as a buffer against anxiety and depressive symptoms in childhood cancer patients (Liu et al., 2023). This study aimed to delineate the factors influencing spiritual well-being. These research findings collectively support the notion that positive childhood experiences may serve as predictors of individuals' spiritual well-being.

Positive childhood experiences (PCEs), such as nurturing relationships and safe environments, contribute to healthy development and well-being (Sege & Browne, 2017), and are associated with lower risks of loneliness, anxiety, and depression as well as increased life satisfaction, self-rated health, and purpose in life (Xu et al., 2022), as well as decreased chances of having adult mental health issues, being in fair or poor health, and getting any physical or mental health ailment at any age (Huang et al., 2023). According to Reich et al. (2010), positive childhood experiences significantly influence various aspects of healthy personality development, with their intensity and duration positively linked to overall psychological well-being. Furthermore, early positive experiences, coupled with quality early childhood education, aid in healthy brain development and stress reduction (Kim & Lee, 2017). Positive childhood experiences serve as buffers against toxic stress and promote health and development (Burstein et al., 2021). Moreover, children experienced to positive childhood experiences, such as engagement in extracurricular activities, community volunteering, mentorship, resilience within families, safe neighborhoods, and supportive caregivers, have higher odds of flourishing (Crouch et al., 2023). Bethell et al. (2019) found a link between higher positive childhood experiences (PCEs) and reduced adulthood depression. Positive childhood experiences have long-term effects on well-being (Richards & Huppert, 2011). They are associated with better outcomes in later years (Bethell et al., 2019; Kocatürk & Çiçek, 2021). Moreover, positive childhood experiences contribute to spiritual well-being by nurturing spiritual potential (King, 2013). This link is associated with reduced psychopathology, stronger friendships, better health, and enhanced well-being (Bożek et al., 2020; King, 2013; Kosarkova et al., 2020). Therefore, positive early experiences may therefore be indicative of spiritual well-being. Spiritual well-being may also be influenced by several factors, like psychological flexibility and meaning-based coping.

### Psychological Flexibility and Meaning-Based Coping as the Mediators

Psychological flexibility, as defined by Gloster et al. (2011), involves fully engaging with the present moment and adapting behavior according to chosen values. Therapies such as Acceptance and Commitment Therapy (ACT) require certain skills in order to work. These include defusion, acceptance, present-moment awareness, strong self-awareness, values, and committed action (Hayes, 2004; Whiting et al., 2017). Psychological flexibility, a multifaceted construct encompassing cognitive, psychological, affective, and behavioral dimensions (Ben-Itzhak et al., 2014), significantly enhances psychological well-being by enabling individuals to adapt to various life demands (Kashdan & Rottenberg, 2010). High psychological flexibility associated with improved adjustment to academic life, well-being, daily goal pursuit, adaptive stress responses, better relationships, and reduced negative affect (Bi & Li, 2021; Cherry et al., 2021; Čekrlija & Schermer, 2024; Kashdan et al., 2020; Twiselton et al., 2020). Moreover, it mitigates psychological symptoms following early life trauma, underscoring its critical role in adaptive functioning, well-being, life satisfaction, and mental health (Lucas & Moore, 2019; Richardson & Jost, 2019).

Positive childhood experiences and psychological flexibility are closely linked, emphasizing early life's role in adaptive behavior and emotional regulation (Kashdan & Rottenberg, 2010). Psychological flexibility, distinct from negative emotionality, is associated with reduced distress and improved well-being (Browne et al., 2022; Kashdan et al., 2020). Positive childhood experiences nurture psychological flexibility, facilitating adaptation to various situations (Matos et al., 2017; Redican et al., 2022). Moreover, higher psychological flexibility in children has been linked to enhanced cooperation with peers, engagement in turn-taking behaviors, and proficiency in verbalizing non-task related topics (Bonino & Cattelino, 1999). Furthermore, positive peer relations during childhood and adolescence have been identified as predictors of increased expressive flexibility in later life (Wang & Hawk, 2020). This underscores the long-term impact of early social experiences on the development of psychological flexibility and underscores the importance of fostering positive environments for children's socioemotional growth and adaptive functioning.

In another study, it was found that psychological flexibility enhances spiritual well-being through engaging in values-based behaviors and cultivating present-moment awareness and openness to events (Borges et al., 2022). Processes associated with psychological flexibility have been applied to alleviate spiritual suffering within the context of moral injury, promoting an open, aware, and engaged approach (Borges et al., 2022). Additionally, Jones et al. (2019) demonstrated that mindfulness meditation enhances coping flexibility, which associated with spiritual well-being. Higher degrees of well-being are linked to more strong emotional reactions, regardless of valence, suggesting psychological flexibility enhances spiritual well-being (Klein et al., 2023). Conversely, individuals lacking positive childhood experiences may experience a decrease in psychological flexibility (Hayes, 2023; Makriyianis et al., 2019), with this psychological inflexibility found to negatively impact spiritual well-being (Scalora et al., 2020). Furthermore, adverse childhood experiences (ACEs), as noted by Makriyianis et al. (2019), have been associated with diminished psychological flexibility and heightened inflexibility, consequently elevating susceptibility to anxiety and depression. It has also been posited by Leung and Pong (2021) that affective disorders such as anxiety and depression may exert adverse effects on spiritual well-being.

An important factor linking positive childhood experiences with spiritual well-being is known as meaning-based coping. This approach involves positively reassessing and reinterpreting stressful situations, focusing on personal growth and effectively managing challenges (Park & Folkman, 1997; Wenzel et al., 2002). This coping style enhances psychological resilience and reduces dysfunctional responses to chronic stress (McEwen, 1998). Individuals using meaning-based coping typically display higher levels of resources and confidence (Holahan & Moos, 1987). Positive childhood experiences, such loving parents and the retention of happy memories, are linked to the adoption of healthy coping mechanisms and overall well-being later in life, according to research findings (Belpame et al., 2020; Moran et al., 2018). Positive childhood experiences impact coping strategies by influencing emotional regulation, emotional expression, and emotional avoidance (Perry & Cuellar, 2021). These experiences bolster coping efforts and reinforce emotional and mental health outcomes among university students (Hanson et al., 2022). Additionally, deriving positive meaning from childhood sexual abuse experiences can lead to reduced isolation and improved interpersonal relationships (Wright et al., 2007).

Research has demonstrated the important function that meaning-based coping, mediated and moderated by the quantity of mental health issues, in terms of improving well-being (Ellis et al., 2016; Guo et al., 2013; Sanchez-Ruiz et al., 2021). Meaning-based coping strategies, integrating spiritual elements, associated with better psychological adjustment and well-being (Park, 2017), suggesting their potential in bolstering mental health and spiritual well-being during adversity. Studies highlight the strong association among meaning-based coping, spiritual well-being, and a high standard of life as a whole which functions as a mediator of stress effects and fosters favorable outcomes for mental health (Arslan & Yıldırım, 2021; Garduño-Ortega et al., 2021). These coping mechanisms also influence spiritual well-being, quality of life, and coping strategies among those with oncological conditions (Whitford & Olver, 2012). Furthermore, heightened spiritual well-being and higher levels of meaning-based coping function as buffers against the negative impacts of pandemic-related stress on young adults' subjective well-being (Arslan & Yıldırım, 2021). Positive reinterpretation and religious engagement, part of meaning-making coping strategies, significantly contribute to spiritual quality of life among palliative care nurses (Desbiens & Fillion, 2007). In summary, meaning-based coping plays a vital role in improving mental health and spiritual well-being across diverse populations.

## The Present Study

Based on the reviewed literature, there is substantial evidence that having a happy childhood is associated with psychological flexibility, which is a major predictor of spiritual well-being. Additionally, higher degrees of spiritual well-being are linked to psychological flexibility and meaning-based coping. Understanding these connections is crucial, so our study focuses on psychological flexibility and meaning-based coping as potential mediators between positive childhood experiences and spiritual well-being. In order to investigate this, we carried out a cross-sectional survey with a focus on the contributions psychological flexibility and meaning-based coping made to the connection between spiritual well-being and positive childhood experiences. This study examined whether psychological flexibility and meaning-based coping are serially mediated mediators in the association between spiritual well-being and positive childhood experiences, in accordance with these theoretical conclusions and prior research results. The ensuing hypotheses (H) were put forth:

*H1*: The association between spiritual well-being and positive childhood experiences is mediated by psychological flexibility.

*H2*: The association between spiritual well-being and positive childhood experiences is mediated by meaning-based coping.

*H3*: psychological flexibility and meaning-based coping act as serial mediators in the relationship between spiritual well-being and positive childhood experiences.

## Methods

### Participants and Procedure

1061 participants were chosen for the study from 75 Turkish cities. With 87 (8.2%) males and 974 (91.8%) females, the participants' mean age was 39.38 years (SD = 8.82, range = 18–66). Undergraduate students made up 44.0% of the participants, followed by those with a graduate degree (11.1%), high school graduates (22.7%), and elementary school graduates (17.2%). Of the participants, 52% were jobless, 42.1% were employed, and the remaining individuals were students. Of the participants, 225 (21.2%) were single and 836 (78.8%) were married. Perceived socioeconomic level was reported by participants as low (17.3%), medium (74.4%), or high (8.3%). Table 1 displays the study participants' demographic information.

**Table 1:** Participants’ characteristics

Variable	Frequency	%
*Gender*		
Female	974	91.8
Male	87	8.2
*Educational status*		
Primary School	182	17.2
High School	294	27.7
University	467	44.0
Master/Ph.D	118	11.1
*Working Status*		
Student	62	5.8
Public sector employee	278	26.2
Private sector employee	169	15.9
Unemployed	552	52
*Marital status*		
Single	225	21.2
Married	836	78.8
*Perceived Socio*-*Economic Status*		
Poor	184	17.3
Medium	789	74.4
Good	88	8.3

Participants provided their agreement to participate through an online survey (Google Forms). Announcements in the classroom, personal invites, and posts on social media were used to entice participants to participate in the study. Convenience sampling was the method employed, and the data collection took place in March 2024. There were no incentives or awards offered, and participation was completely voluntary. The work received ethical approval from the Scientific Research and Ethical Review Board of Yıldız Technical University (REF: 20240402834). The informed consent form, which the participants had to fill out before beginning the study, stated that they might withdraw from it at any time. After that, they were required to complete surveys about their demographics (age, gender, level of education, economic status, etc.) as well as positive childhood experiences, psychological flexibility, meaning-based coping, and spiritual well-being. In order to verify that no data was missing, participants were motivated to complete every item on the questionnaire.

### Measures

#### Positive Childhood Experiences

A Turkish adaptation of Bethell et al. (2019)'s Positive Childhood Experiences Scale (PCEs) been made (Çiçek & Çeri, 2021). It was employed to evaluate the participants' histories of positive childhood experiences. With responses ranging from virtually never (1) to always (5) on a five-point Likert scale, the PCEs is a seven-item questionnaire. Higher scores suggest higher levels of pleasant childhood experiences. "How often were you able to talk about your feelings with your family?" is one example of a PCE question. Prior research employing PCEs has demonstrated that the scale's Cronbach's alpha, when given to adult Turkish participants, is 0.78 (Çiçek & Çeri, 2021).

#### Psychological Flexbility

Yıldırım and Aziz (2023) adapted the Psy-Flex Scale, which was created by Gloster et al. (2021), for use in the Turkish context. Six things are included in it, such as "I pay attention to all the details of things that are important, beneficial, or meaningful to me." Answers were provided on a 5-point Likert scale with 1 denoting "extremely infrequently" and 5 denoting "very often). The scale yields scores between 6 and 30. Higher scores are indicative of more psychological adaptability. The confirmatory factor analysis results of the Yıldırım and Aziz (2023) investigation are as follows: χ^2^(8) = 35.637, RMSEA (root mean square error of approximation) = 0.082, CFI (comparative fit index) = 0.97, TLI (Tucker-Lewis index) = 0.94, and SRMR (standardized root mean residual) = 0.041. The scale's Cronbach's alpha is 0.80.

#### Meaning-Centered Coping

An version of the meaning-centered coping scale (MCCS; Eisenbeck et al., 2021) was used to fit the Turkish sample (Arslan & Yıldırım, 2021). It was applied to evaluate people's existential positive psychology-based meaning-based coping mechanisms. The MCCS consists of a nine-item questionnaire on a seven-point Likert scale ranging from 1 (I don't agree at all) to 7 (I totally agree). One instance of an item is "I am grateful for my life as it is." Higher meaning-based coping levels are indicated by higher scores. The MCCS in Turkish (Arslan & Yıldırım, 2021) was employed, and the results indicate that it has a verified factor analysis (χ^2^ = 262.20, df = 111, CFI = 0.96, TLI = 0.95, RMSEA = 0.057, and SRMR = 0.035) and strong internal reliability (α = 0.85).

#### Spiritual Well-Being

The five-item Spiritual Well-Being Scale (Arslan & Yıldırım, 2021) is a tool used to assess spiritual well-being among Turkish populations. It was developed based on the FACIT-Sp-12 (Bredle et al., 2011) and includes items such as "I have a productive life," which is rated on a five-point Likert scale from 0 (not at all) to 4 (very much). Greater degrees of spiritual well-being are indicated with greater scores on this one-factor measure, which comprises one reverse item. A thorough statistical analysis demonstrated a strong fit (μ range = 0.55–0.85), a dependable latent construct (H = 0.89), and strong factor loadings (CFI = 0.99, TLI = 0.99, RMSEA (95% CI) = 0.045 (0.00, 0.11), SRMR = 0.025) between the data and the model (Arslan & Yıldırım, 2021).

### Data Analysis

We assessed the mediation of the association between spiritual well-being and positive childhood experiences by psychological flexibility and meaning-based coping using a two-step structural equation modeling technique. For each variable, first descriptive statistics (mean, standard deviation, skewness, and kurtosis) and Pearson correlations were computed. The creation and assessment of a measurement model came next, and structural modeling was completed last. The models' goodness-of-fit was evaluated using the χ^2^ goodness-of-fit statistic, the GFI, the RMSEA, the CFI, and the Adjusted Goodness-of-Fit Index (AGFI). Additionally, the parceling method, employed to mitigate measurement errors in single-factor measurement were integrated into the Structural Equation Model (SEM) (Little et al., 2002). As such, the other mediating variable was divided into three parcels, while the unidimensional constructs of positive childhood experiences (PCEs) and psychological flexibility were each divided into two parcels. Using 5000 bootstrap samples, the mediating variable's significance was evaluated. 95% confidence intervals (CI) were produced for the indirect effects using this method. According to Hayes (2018), indirect effects with bootstrapped 95% CIs excluding zero were considered statistically significant. AMOS Graphics and the IBM Statistical Package for the Social Sciences (SPSS) version 27 were used for all data analysis.

## Results

### Preliminary Analysis

The descriptive statistics, reliabilities, and Pearson's correlation coefficients for the study variables are shown in Table 2. According to Table 2's association analysis, spiritual well-being was positively correlated with all variables. In addition, positive childhood experiences were found to be positively correlated with psychological flexibility and meaning-based coping.

**Table 2:** Descriptive statistics and correlations

Variables	Descriptive statistics and reliabilities						Correlations		
	Mean	SD	Skewness	Kurtosis	α	λ6	1	2	3
1. Spiritual wellbeing	14.37	4.21	− 0.815	0.383	0.81	0.80	–		
2. PCEs	21.81	5.84	− 0.134	− 0.688	0.75	0.75	0.42^**^	–	
3. Psychological flexibility	20.67	4.55	− 0.625	0.290	0.75	0.73	0.50^**^	0.35^**^	–
4. Meaning−based coping	47.00	9.83	− 0.702	0.386	0.84	0.84	0.55^**^	0.31^**^	0.50^**^

^**^ *p* < 0.01

### Structural Equation Modeling

We verified the presumptions first. Skewness and kurtosis values were used to test the normalcy assumption, and acceptable values were established in accordance with George and Mallery's (2010) suggestions (Table 2). Furthermore, it was discovered that the dependability coefficients exceeded the permissible threshold of .70. Durbin-Watson (DW), tolerance, and the variance-inflated factor (VIF) were used to assess multicollinearity. Every tolerance value was greater than .10, and the VIFs were all less than 10. There was no discernible relationship between the residuals, as indicated by the DW value of 2.062. Thus, the residuals and multicollinearity issues were resolved. Consequently, all presumptions were satisfied in line with Field's (2016) recommendations. We started the two-step structural equation modeling process when all presumptions were met. The model measurement was examined first.

**Fig. 1:**
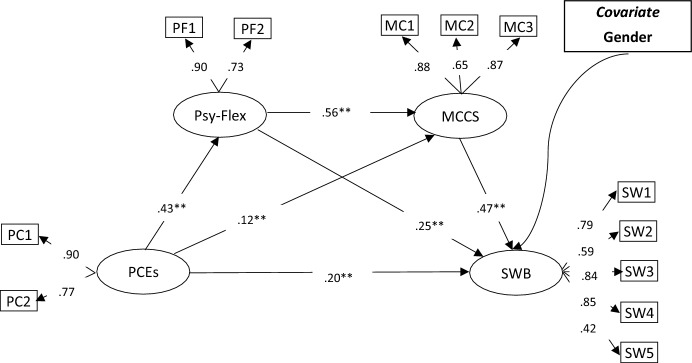
Structural equation modeling for the serial mediation model. **p < .01, PC1 and PC2: parcels of Positive childhood experiences, PF1 and PF2: parcels of psychological flexibility, MC1, MC2, and MC3: parcels of meaning based coping

Twelve observable variables and four latent variables (meaning-based coping, positive childhood experiences, spiritual well-being, and psychological flexibility) made up the measurement model. The measurement model's outcomes show that the model and the data fit each other well: The values of χ^2^(48) = 211.641, χ^2^/df = 4.41, GFI = 0.967, NFI = 0.966, CFI = 0.973, TLI = 0.963, IFI = 0.973, RMSEA = 0.057, SRMR = 0.036. The measurement model's factor loadings ranged from 0.42 to 0.90 (*p* < 0.01). Next, we put the serial mediation idea to the test using structural modeling. Gender was one of the control variables in the model. The model (Fig. 1) has good levels of model fit: χ^2^(59) = 263.167, χ^2^/df = 4.46, GFI = 0.962, NFI = 0.958, CFI = 0.967, TLI = 0.956, IFI = 0.967, RMSEA = 0.057, SRMR = 0.040. According to the paths among the latent factors, positive childhood experiences are associated with psychological flexibility (β = 0.43, *p* < 0.001) and meaning-based coping (β = 0.12, *p* < 0.001). Furthermore, psychological flexibility (β = 0.25, *p* < 0.001) and meaning –based coping (β = 0.47, *p* < 0.001) are associated with spiritual well-being.

The partial mediation model was evaluated and contrasted with the full mediation model in order to test the hypotheses. With χ^2^/df = 4.409, CFI = 0.973, NFI = 0.966, TLI = 0.963, GFI = 0.967, RMSEA = 0.057, SRMR = 0.036, AIC = 327.167, and ECVI = 0.309, the partial mediation model showed a respectable fit to the data. The fit of the entire model produced lower fit indices (χ^2^/df = 5.103, CFI = 0.960, NFI = 0.951, TLI = 0.948, GFI = 0.960, RMSEA = 0.062, SRMR = 0.048, AIC = 368.196, ECVI = 0.347) in comparison to the partial mediation model. The AIC and ECVI coefficients of the partial mediation model were less than those of the full mediation model, which is why it was chosen. Table 3 also displays the overall and indirect impacts for bootstrapping.

**Table 3:** Standardized bootstrapping coefficients for the model

Model pathways	Coefficient^*^	95% CI	
		Lower	Upper
*Indirect effect*			
PCEs → Psy-Flex → SWB	0.038	0.024	0.057
PCEs → MBC → SWB	0.021	0.009	0.036
PCEs → Psy-Flex → MBC → SWB	0.041	0.030	0.056

*Because the CIs do not cover zero, all the coefficients are significant

*PCEs* positive childhood experiences, *Psy-Flex* psychological flexibility, *MBC* meaning-based coping, *SWB* spiritual well-being, *CI* confidence interval

## Discussion

Spiritual well-being is closely associated with the sense of wholeness, transcendence of boundaries, and overall satisfaction, while also being intertwined with happiness and the exploration of life's meaning. In this regard, spiritual well-being can serve significant functions, such as coping with life's challenges and finding fulfillment in the pursuit of profound purpose, thereby contributing to the attainment of inner peace and the meaningful interpretation of one's life experience. According to the study's findings, the relationship between spiritual well-being and positive childhood experiences is mediated sequentially by psychological flexibility and meaning-based coping.

The link between PCEs and psychological flexibility discovered in this investigation confirms the findings of positive childhood experiences can cultivate psychological flexibility, enabling individuals to adjust to different circumstances (Matos et al., 2017; Redican et al., 2022). According to previous research, individuals who experience positive childhood experiences like parental warmth and collaboration with peers early in life may enhance psychological flexibility (Bonino & Cattelino, 1999; Wang & Hawk, 2020). In addition, the people are able to continue this distinctive feature during their life-long (Wang & Hawk, 2020). Furthermore, research has shown that psychological flexibility is a strong predictor of mental health and that all forms of wellness are positively correlated with happy childhood memories (Fledderus et al., 2010; Gloster et al., 2017; Twiselton et al., 2020). Furthermore, in line with recent research on the connection between psychological flexibility and general well-being (Browne et al., 2022; Kashdan et al., 2020), our study shows that psychological flexibility is associated with higher levels of spiritual well-being.

Another significant conclusion of this study is that meaning-based coping mediates the relationship between positive childhood experiences and spiritual well-being, which is consistent with previous research showing that PCEs can promote meaning-based coping (Belpame et al., 2020; Moran et al., 2018). PCEs, as highlighted by Perry and Cuellar (2021), shape coping strategies by impacting emotional regulation, expression, and avoidance, consequently bolstering coping efforts and reinforcing emotional and mental health outcomes as noted by Hanson et al. (2022). Furthermore, it is noteworthy that children who derive positive meaning from experiences of childhood sexual abuse exhibit a remarkable tendency towards decreased isolation and enhanced interpersonal relationships (Wright et al., 2007). Consistent with earlier studies (Arslan & Yıldırım, 2021; Garduño-Ortega et al., 2021) that revealed meaning-based coping to be positively connected with spiritual well-being, our analysis demonstrates that meaning-based coping strongly predicts spiritual well-being.

Ultimately, this study verified the serial mediation model, which involves psychological flexibility and meaning-based coping in the association between spiritual well-being and pleasant childhood experiences. Prior studies have also looked into the connection between psychological flexibility and meaning-based coping, people who have higher levels of psychological flexibility and use coping strategies focused on meaning-based tend to handle not only psychological challenges such as trauma, abuse, and childhood maltreatment but also daily stressor more effectively (DeBeer et al., 2017; Finkelstein-Fox et al., 2019; Türk et al., 2024). Furthermore, employing meaning-based coping techniques is essential for amplifying the benefits of psychological flexibility (Avsec et al., 2022). Thus, rather than using an adaptive coping strategy, those have a tendency to rely on meaning and other practical tactics. This study's serial mediation approach sheds light on the intricate relationships that exist between positive childhood experiences, meaning-based coping, psychological flexibility, and spiritual well-being.

Overall, this study's findings emphasize how critical it is to comprehend the processes by which happy childhood memories might influence spiritual well-being. Important moderators of this relationship are psychological flexibility and meaning-based coping, which may have consequences for mental health therapies meant to enhance psychological well-being in general and spiritual well-being in particular. However, additional investigation is required to enhance our comprehension of these processes and their possible uses in medical environments.

### Implications

The study's conclusions have significant ramifications for comprehending how positive childhood experiences can shape individuals' long-term well-being into adulthood. Positive experiences during childhood, such as nurturing and supportive environments, can enhance psychological flexibility and the ability to find meaning in life's challenges through meaning-based coping. This leads to higher states of spiritual well-being. Caregivers need to understand how much their actions affect a child's whole life and take steps to guarantee that children grow up in a safe and nurturing environment.

To address the issue of early childhood and foster well-being, the Early Care and Education (ECE) programs play a crucial role. These programs, as highlighted by Roberts et al. (2016) and Whitaker et al. (2015), emphasize providing quality care and educational opportunities for young children. By offering a nurturing and stimulating environment, ECE programs aim to support children's holistic development. Moreover, they often incorporate health and nutrition components, ensuring children's physical well-being alongside their cognitive and social growth. Through collaborative efforts involving educators, caregivers, and policymakers, ECE programs contribute significantly to promoting the overall well-being of young children.

Moreover, combining meditation with psychotherapy can enhance spiritual well-being through the cultivation of self-awareness and the facilitation of emotional and cognitive transformations (Kutz et al., 1985). These treatments have demonstrated efficacy in mostly Muslim nations such as Iran and Indonesia, and given Türkiye's cultural parallels with those societies, there is potential for their implementation among Turkish communities. Such spiritual interventions offer an opportunity for promoting positive childhood experiences.

In light of Türkiye's predominantly collectivist society, it can be advantageous to promote collective involvement in spiritual or religious activities. Establishing nurturing spaces where people can participate in spiritual activities collectively could promote a feeling of inclusion and wellness. Establishing such surroundings for individuals in need is therefore imperative.

### Limitations

The study has a number of significant drawbacks. First of all, causal relationships are harder to establish due to the cross-sectional design it employs. In order to more thoroughly examine the connections between these variables, experimental or longitudinal study designs should be used in future studies. Second, the bulk of the participants were adults, and they came from a variety of Turkish provinces, each at a different developmental level. When extrapolating the study results to a larger population, these variances should be taken into account. Finally, using self-report measures to gather data raises questions regarding possible biases including social desirability and memory recall. Future research could solve this by incorporating observational methods or conducting interviews to provide a more detailed and accurate assessment of the factors being examined.

## Conclusion

To sum up, this research provides insightful information about spiritual health in the context of Turkish culture. The results are consistent with the suggested model, which holds that the association between spiritual well-being and positive childhood experiences is mediated by psychological flexibility and meaning-based coping, notwithstanding the aforementioned restrictions.

## Data Availability

Data will be will be provided upon request.
